# Influence of carbon chain length on the synthesis and yield of fatty amine-coated iron-platinum nanoparticles

**DOI:** 10.1186/1556-276X-9-306

**Published:** 2014-06-17

**Authors:** Robert M Taylor, Todd C Monson, Rama R Gullapalli

**Affiliations:** 1 Department of Pathology, University of New Mexico, Room 308, MSC06-4840, Albuquerque, NM 87131, USA; 2 Nanoscale Sciences Department, Sandia National Laboratories, P.O. Box 5800, MS-1415, Albuquerque, NM 87185, USA; 3 Department of Chemical and Nuclear Engineering, University of New Mexico, Room 333A, MSC08-4640, Albuquerque, NM 87131, USA

**Keywords:** Iron-Platinum nanoparticles, SIPPs, Magnetic nanoparticles, Nanoparticle synthesis, Nanoparticle characterization, Cancer biology

## Abstract

Iron oxide nanoparticles are among the most widely used and characterized magnetic nanoparticles. However, metal alloys such as superparamagnetic iron-platinum particles (SIPPs), which have better magnetic properties, are receiving increased attention. Scalable techniques to routinely synthesize SIPPs in bulk need further study. Here, we focus on the role played by the fatty amine ligand in the formation of the bimetallic FePt nanocrystal. More specifically, we compare the effect of varying lengths of fatty amine ligands on the shape, structure, uniformity, composition, and magnetic properties of the SIPPs. We synthesized SIPPs by employing a ‘green’ thermal decomposition reaction using fatty amine ligands containing 12 to 18 carbons in length. Greater fatty amine chain length increased the polydispersity, particle concentration, iron concentration, and the stability of the SIPPs. Additionally, longer reflux times increased the diameter of the particles, but decreased the iron concentration, suggesting that shorter reaction times are preferable. Fourier transform infrared spectroscopy of the SIPPs indicates that the ligands are successfully bound to the FePt cores through the amine group. Superconducting quantum interference device magnetometry measurements suggest that all of the SIPPs were superparamagnetic at room temperature and that SIPPs synthesized using tetradecylamine had the highest saturation magnetization. Our findings indicate that the octadecylamine ligand, which is currently used for the routine synthesis of SIPPs, may not be optimal. Overall, we found that using tetradecylamine and a 30-min reflux reaction resulted in optimal particles with the highest degree of monodispersity, iron content, stability, and saturation magnetization.

**PACS:**

81.07.-b; 75.75.Fk; 61.46.Df

## Background

Magnetic nanoparticles have found a multitude of applications in biomedical research, such as radiological contrast agents, magnetic hyperthermia treatment modalities, nanomedicine, and targeted drug delivery of cancer agents (e.g., paclitaxel) to name a few [1-4]. Magnetic nanoparticles are mainly classified into three different categories: (a) metal oxide nanoparticles such as iron oxides, which are not very strong magnetically, but stable in solution [5]; (b) metallic nanoparticles which are magnetically strong but unstable in solution [5]; and (c) metal alloys such as iron-platinum nanoparticles and cobalt-platinum nanoparticles which have high magnetic properties and are also stable in solution [5]. In addition to biocompatibility, biomedical applications require the nanoparticles to be stable in harsh ionic *in vivo* environments such as human sera and plasma solutions. The nature of the magnetic nanoparticle surface determines the important properties such as biocompatibility and stability in solutions.

Magnetic nanoparticles can be synthesized through a multitude of methods including alkaline solution precipitation, thermal decomposition, microwave heating methods, sonochemical techniques, spray pyrolysis, and laser pyrolysis to name a few [1,4,6,7]. Of all the methods, thermal decomposition of organometallic iron in organic liquids provides the most reliable means of nanoparticle synthesis with good control over the size and shape of the particles [1,6,7]. Thermal decomposition methods yield particles that are more crystalline and uniform in shape ranging from 3 to 60 nm in diameter [1,4,7]. In contrast, alkaline precipitation in aqueous solutions creates particles that have a much higher range of variability in size and shapes of the nanoparticles [1,7]. The shape and properties of the synthesized particles are highly dependent on the starting material used in the alkaline precipitation method (i.e., nitrates vs. chlorides vs. sulfates) [7]. However, thermal decomposition suffers from the drawback of using relatively toxic precursors in the syntheses. Thermal decomposition methods use toxic metallic precursors such as iron pentacarbonyl (Fe(CO)_5_) and other organic solvents for the process of synthesis [1,4,7]. There is much interest currently in alternative methods of nanoparticle synthesis, which use relatively non-toxic starting precursors and are environmentally friendly. It is now possible to prepare nanoparticles using much less toxic chemical precursors, such as iron fatty acids [2,8-10]. These so-called green synthesis methods are much less toxic and can produce relatively stable and uniform magnetic nanoparticles [8,10]. Superparamagnetic iron-platinum particles (SIPPs) produced using such methods are seen to maintain their relative stability in solutions [2,8,9]. Uniformity of size and shape of nanoparticles are important for issues related to biocompatibility as a widely varying size range may lead to non-uniform behavior of the nanoparticles both *in vitro* and *in vivo*[11].

The general reaction for the synthesis of magnetic nanoparticles using a green method of synthesis is described as follows. The iron precursor of the reaction is in the form of iron fatty acids (Fe-fatty acid). The second component of the bimetallic nanoparticle is a platinum precursor in the form of platinum acetylacetonate or Pt(acac)_2_. The solvent of the reaction is octadecene (ODE) or tetracosane (TCA). A fourth component of the reaction is the use of fatty amines and fatty acids as ligands. Fatty amines, in the form of octadecylamine (ODA), are carbon-18 single chain fatty amines that play a critical role in the stabilization of the nanocrystal in the early stages of synthesis [10]. Moreover, fatty amines can act as both the solvent and the ligand, reducing the number of chemicals needed to produce the alloy nanocrystals. In this report, we focus on the open question of the role played by the fatty amine in the formation of the bimetallic FePt nanocrystal. More specifically, we compare the effect of varying lengths of fatty amine ligands on the shape, structure, uniformity, composition, and magnetic properties of the synthesized magnetic FePt nanoparticles.

## Methods

### Materials used for synthesis

Iron nitrate nonahydrate (Fe(NO_3_)_3_ · 9H2O) and Pt(acac)_2_ were purchased from Sigma (St. Louis, MO, USA). Additionally, all of the ligands including ODA, 1-hexadecylamine (HDA), 1-tetradecylamine (TDA), and 1-dodecylamine (DDA) were purchased from Sigma (St. Louis, MO, USA) and had carbon chain lengths of 18:0, 16:0, 14:0, and 12:0, respectively, and used without further modifications. A temperature controller (model 210-J) and heating mantle were purchased from J-KEM Scientific, Inc. (St. Louis, MO, USA). The thermocouple (type 316 SS probe) was purchased from McMaster-Carr (Los Angeles, CA, USA). All glassware was purchased from VWR (Radnor, PA, USA).

### Synthesis method

SIPPs, stabilized with the various fatty amines, were synthesized using slight modifications of a procedure we have described previously [2,8,9]. Briefly, 1.0 mmol of Fe(NO_3_)_3_ · 9 H_2_O and 1.0 mmol of Pt(acac)_2_ were combined with 12.5 mmol ODA in a 25-mL three-neck round bottom flask fitted with a reflux condenser. Alternately, HDA, TDA, or DDA were used instead of ODA. Refluxing (340°C to 360°C) was continued for either 30 or 60 min, and then the reaction flask was removed from the heat and allowed to cool to room temperature. The resulting black particles were collected in approximately 80 mL of hexane. The 20-mL aliquots of the collected particles, in hexane, were placed in 50-mL conical tubes and diluted with 30 mL of ethanol (EtOH). The suspensions were then centrifuged at 1,462 × *g* for 10 min. The solution was discarded and the pelleted particles were again suspended in 20 mL hexane. The resuspended particles were then equally divided in the two 50-mL conical tubes, diluted with 40 mL of EtOH, and centrifuged at 1,462 × *g* for 5 min. The EtOH serves to wash the excess ligand from the nanoparticle solutions. Finally, the solution was discarded, and the purified SIPP pellets were collected in a total volume of 20 mL hexane and stored at room temperature in glass scintillation vials.

### Characterization methods

Transmission electron microscopy (TEM) was used to quantify the size and polydispersity of the SIPPs, as well as to determine the morphology. A 5-μL aliquot of particles was applied to a 7.0-nm-thick carbon-coated copper grid purchased from Dr. Stephen Jett (University of New Mexico, Albuquerque, NM, USA) and allowed to dry. The samples were then imaged on a Hitachi 7500 TEM with an acceleration voltage of 80 kV. The resultant TEM images were analyzed using ImageJ Software [12]. At least 200 particles were counted, per sample. A region of interest (ROI) was drawn around each particle, and the mean Feret diameters and standard deviations were calculated.

The compositions of the various SIPPs were investigated using thermogravimetric analysis (TGA). The hexane was allowed to evaporate from the aliquots of SIPPs in the hood overnight, and portions of the dried SIPPs were then placed in TGA crucibles (Robocasting Enterprises LLC, Albuquerque, NM, USA) after taring. Weight loss profiles of the dried samples were measured against a reference crucible using an SDT Q600 TGA/DSC (TA Instruments, New Castle, DE, USA) under a flow of nitrogen. The ligand and naked FePt content were quantified by measuring the change in mass as the temperature was raised from room temperature to 900°C at a 20°C per minute ramping rate. The derivative weight percent per degrees Celsius was also plotted versus temperature.

Fourier transform infrared spectroscopy (FTIR) was employed to determine if the fatty amine ligands were bound to the iron-platinum alloys. The hexane was allowed to evaporate from aliquots of the SIPPs in the hood overnight, and portions of the dried SIPPs were then applied to the surface of an alpha FTIR fitted with a Bruker platinum-attenuated total reflectance (ATR) probe (Bruker, Billerica, MA, USA). Data was analyzed using OPUS software (Bruker, Billerica, MA, USA).

The metal content and iron to platinum stoichiometry of the different samples were measured using a PerkinElmer Optima 5300 DV (Waltham, MA, USA) inductively coupled plasma-optical emission spectroscopy (ICP-OES) instrument. The samples were digested in a 1:2 (*v*/*v*) mixture of nitric and hydrochloric acids in PDS-6 pressure digestion systems (Loftfields Analytical Solutions, Neu Eichenberg, Germany) and were then made up to volume and mixed, and impurities were pelleted by centrifugation. The samples were analyzed using the recommended wavelength for both iron and platinum. Analysis was performed in an axial mode to improve detection limits. A blank and set of calibration standards were used to establish a three-point calibration curve. Calibration verification samples were analyzed prior to analyzing samples. Analyte peaks were examined, and peak locations and background points were adjusted for optimum recoveries.

The saturation magnetizations and blocking temperatures of the samples were measured using a Quantum Design MPMS-7 superconducting quantum interference device (SQUID) magnetometry. Aliquots (100 μL) of the samples were applied to Qtips® cotton swabs (Unilever, Englewood Cliffs, NJ, USA) and allowed to dry. The samples were then scanned using temperature sweeps up to 340 K by zero-field cooling the sample and measuring the magnetic moment as a function of temperature in the presence of a 1-mT magnetic field during heating and subsequent cooling. The values for the blocking temperatures were then extrapolated from the peak location in the resultant zero-field cooled (ZFC) curve. Similarly, the applied magnetic field was swept from −5 to 5 T at room temperature (293.15 K) to measure the magnetic moment as a function of applied field. The data was fit over a range of points approaching 5 T to determine the saturation magnetizations of the samples. After the SQUID magnetometry measurements were completed, the cotton swab samples were digested in acid and the iron content was quantified using ICP-OES, as described above. The iron concentration was then used to calculate the mass magnetizations of each sample.

## Results and discussion

**Figure 1:**
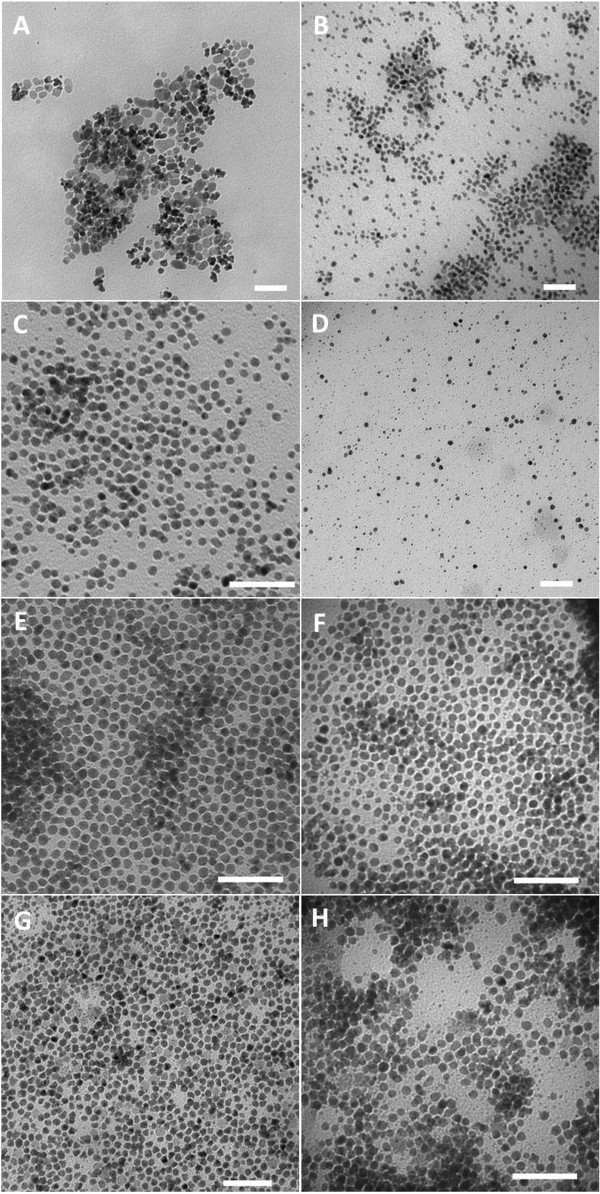
**TEM images of SIPPs.** TEM images of SIPPs synthesized using ODA **(A,B)**, HDA **(C,D)**, TDA **(E,F)**, and DDA **(G,H)**. SIPPs were allowed to reflux for either 30 min (left column) or 60 min (right column). Scale bars are 50 nm.

SIPPs were successfully synthesized using all four of the fatty amines. Figure 1 shows TEM images of the SIPPs synthesized using ODA, HDA, TDA, and DDA and refluxed for either 30 or 60 min. The TEM images indicate that the particles became less polydispersed with decreasing chain length, as the particles synthesized using the carbon-14 chain amine (TDA) and the carbon-12 chain amine (DDA) become more uniform in size compared to the SIPPs synthesized using the longer-chained ODA and HDA. We measured the diameter and standard deviation using ImageJ software. Table 1 lists the diameters and standard deviations of the different SIPPs. We also calculated the coefficient of variation in the diameters, a measure of polydispersity, and included this is Table 1. It is clear that the lowest coefficient of variation and, therefore, lowest polydispersity were found for the SIPPs synthesized with the TDA and DDA, in agreement with the qualitative analysis of the TEM images. It should be noted, though, that the DDA used to synthesize the DDA-SIPPs was corrosive and corroded part of the inside of the septum used with the reflux apparatus. For this reason, the SIPPs synthesized with TDA (TDA-SIPPs) appear to be a better option, striking an appropriate balance between the safety aspects of synthesis and delivering the lowest polydispersity of the final nanoparticles synthesized.

**Table 1:** Structural characterization of SIPPs

**Value**	**Description**	**Units**	**18SIPP30**	**18SIPP60**	**16SIPP30**	**16SIPP60**	**14SIPP30**	**14SIPP60**	**12SIPP30**	**12SIPP60**
*L*	Chain length	-	18	18	16	16	14	14	12	12
*t*	Reflux time	min	30	60	30	60	30	60	30	60
*d*	Diameter	nm	11.29 ± 3.22	7.20 ± 1.81	6.83 ± 1.34	5.14 ± 2.13	7.34 ± 1.22	6.14 ± 1.67	7.92 ± 1.29	7.34 ± 1.12
CV	Coefficient of variation	%	28.49	25.1	19.6	41.5	16.6	27.3	16.3	15.3
*V*_p_	Particle volume	cm^3^	1.95 × 10^−18^	1.96 × 10^−19^	1.67 × 10^−19^	7.12 × 10^−20^	2.07 × 10^−19^	1.21 × 10^−19^	2.60 × 10^−19^	2.07 × 10^−19^
*S*	Surface area	cm^2^	7.55 × 10^−12^	1.63 × 10^−12^	1.47 × 10^−12^	8.31 × 10^−13^	1.69 × 10^−12^	1.19 × 10^−12^	1.97 × 10^−12^	1.69 × 10^−12^
*C*_p_	Suspension concentration	mg/mL	9.33 ± 0.70	18.30 ± 0.00	5.36 ± 0.43	4.92 ± 0.13	4.29 ± 0.47	5.68 ± 0.43	3.22 ± 0.25	4.74 ± 0.40
*C*_Fe_	Iron concentration	mg/mL	0.369 ± 0.001	0.315 ± 0.0009	0.163 ± 0.001	0.151 ± 0.001	0.214 ± 0.00007	0.210 ± 0.001	0.080 ± 0.0004	0.139 ± 0.0007
*C*_Pt_	Platinum concentration	mg/mL	0.914 ± 0.001	1.068 ± 0.0007	0.332 ± 0.002	0.534 ± 0.002	0.583 ± 0.0003	0.692 ± 0.001	0.205 ± 0.0002	0.463 ± 0.0007
*N*^a^_Fe_	Iron atoms in 1.0 mL	-	3.98 × 10^18^	3.40 × 10^18^	1.76 × 10^18^	1.63 × 10^18^	2.31 × 10^18^	2.26 × 10^18^	8.63 × 10^17^	1.50 × 10^18^
*N*_SIPP_	Nanoparticles per milliliter	SIPP/mL	1.04 × 10^14^	1.02 × 10^15^	4.96 × 10^14^	1.37 × 10^15^	5.90 × 10^14^	1.08 × 10^15^	1.71 × 10^14^	4.21 × 10^14^
A_Fe_	Atomic percent Fe	at.%	58.5	50.8	63.1	49.8	56.2	51.4	57.7	51.1
A_Pt_	Atomic percent Pt	at.%	41.5	49.2	36.9	50.2	43.8	48.6	42.3	48.9
Fe/Pt	Fe/Pt stoichiometry	-	1.41	1.03	1.71	0.99	1.28	1.06	1.36	1.05
*ρ*_FePt_	Density	g/cm^3^	14.0	14.0	14.0	14.0	14.0	14.0	14.0	14.0
*m*^p^_FePt_	Mass per particle	g	2.73 × 10^−17^	2.74 × 10^−18^	2.34 × 10^−18^	9.97 × 10^−19^	2.90 × 10^−18^	1.70 × 10^−18^	3.65 × 10^−18^	2.90 × 10^−18^
*N*^a^_FePt_	Total Fe + Pt atoms per particle	-	65,453	6,573	5,611	2,391	6,964	4,076	8,749	6,964
*N*^p^_Fe_	Iron atoms per particle	-	38289.9	3339.1	3540.5	1190.9	3913.8	2095.3	5048.1	3558.6
*N*^p^_Pt_	Platinum atoms per particle	-	27162.9	3234.0	2070.4	1200.5	3050.3	1981.1	3700.8	3405.4
*W*_L_	Weight percent ligand	wt.%	27.2	50.0	52.9	42.5	43.2	33.7	34.4	41.7
*W*_FePt_	Weight percent naked FePt	wt.%	72.8	50.0	47.1	57.5	56.8	66.3	65.6	58.3
*N*^p^_L_	Number of ligands per mg SIPPs	Ligand/mg SIPP	6.08 × 10^17^	1.12 × 10^18^	1.32 × 10^18^	1.06 × 10^18^	1.22 × 10^18^	9.52 × 10^17^	1.12 × 10^18^	1.36 × 10^18^
*I*_FeO*x*_	Intensity of iron oxide peak (TGA)	Deriv. wt.% °C	0.091	0.068	0.033	0.047	0.054	0.019	0.000	0.000

**Figure 2:**
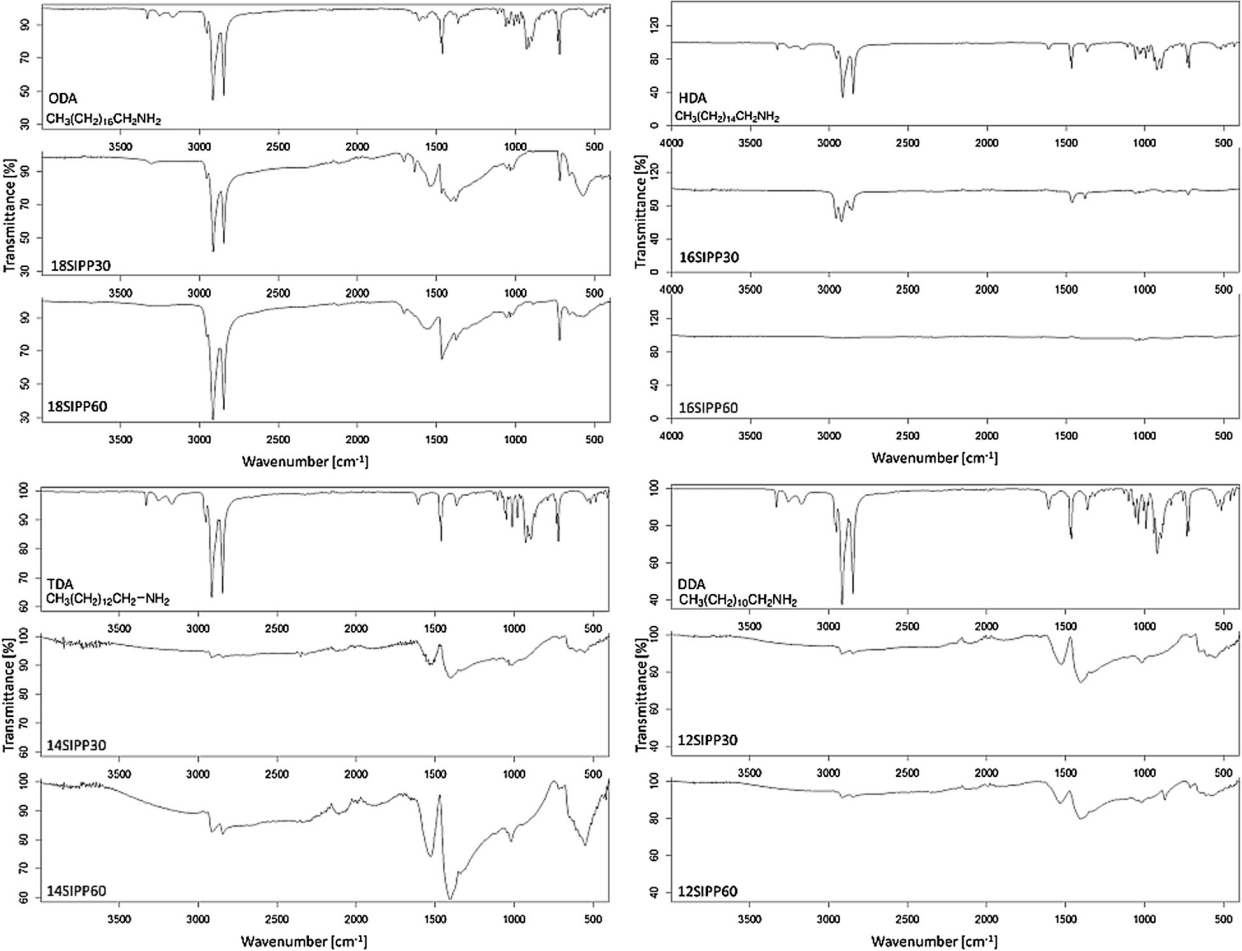
**FTIR spectrographs of SIPPs and fatty amines.** FTIR spectrographs of SIPPs synthesized using ODA (top left), HDA (top right), TDA (bottom left), and DDA (bottom right). Please refer to the text for more details.

We next examined whether the fatty amine ligands were bound to the SIPP alloy cores, using FTIR. Figure 2 shows the spectrograms of each of the fatty amines alone, as well as the particles synthesized using the various ligands with either a 30- or 60-min reflux time. The peaks at approximately 900 and approximately 3,350 to 3,500 cm^−1^ corresponding to the amine stretching and wagging are clearly visible in each of the spectra of the ligands alone. In contrast, these amine peaks in the FT-IR spectra disappear in all spectra of SIPPs. This suggests that the fatty amines were all bound to the surface of the SIPP alloy surface through the amine groups, regardless of which ligand was used or the amount of time the reaction was allowed to reflux. It has also been suggested [13] that and Fe-O stretch can be observed at approximately 580 to 600 cm^−1^. We noticed a broad peak in all of the SIPP spectra, except that for the DDA-SIPPs, suggesting that some iron oxide contamination may also be present in the samples.

**Figure 3:**
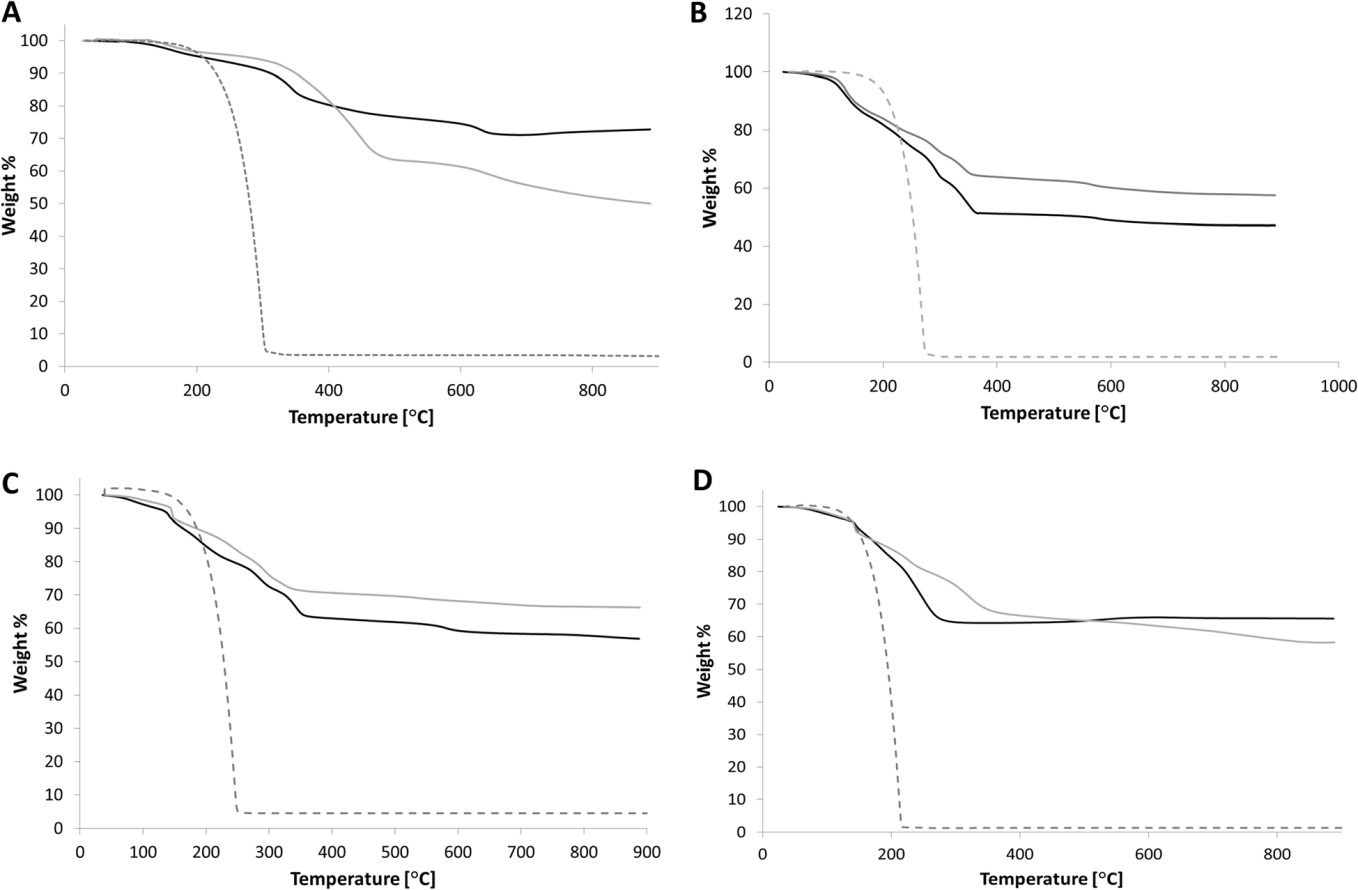
**TGA thermograms of SIPPs and fatty amines.** TGA thermograms of the SIPPs synthesized using ODA **(A)**, HDA **(B)**, TDA **(C)**, and DDA **(D)**. Dotted line = ligand only, black line = 30-min reflux, and gray line = 60-min reflux. The weight percent of ligands and naked alloy, as well as quantification of the number of bound ligands, is listed in Table 1.

**Figure 4:**
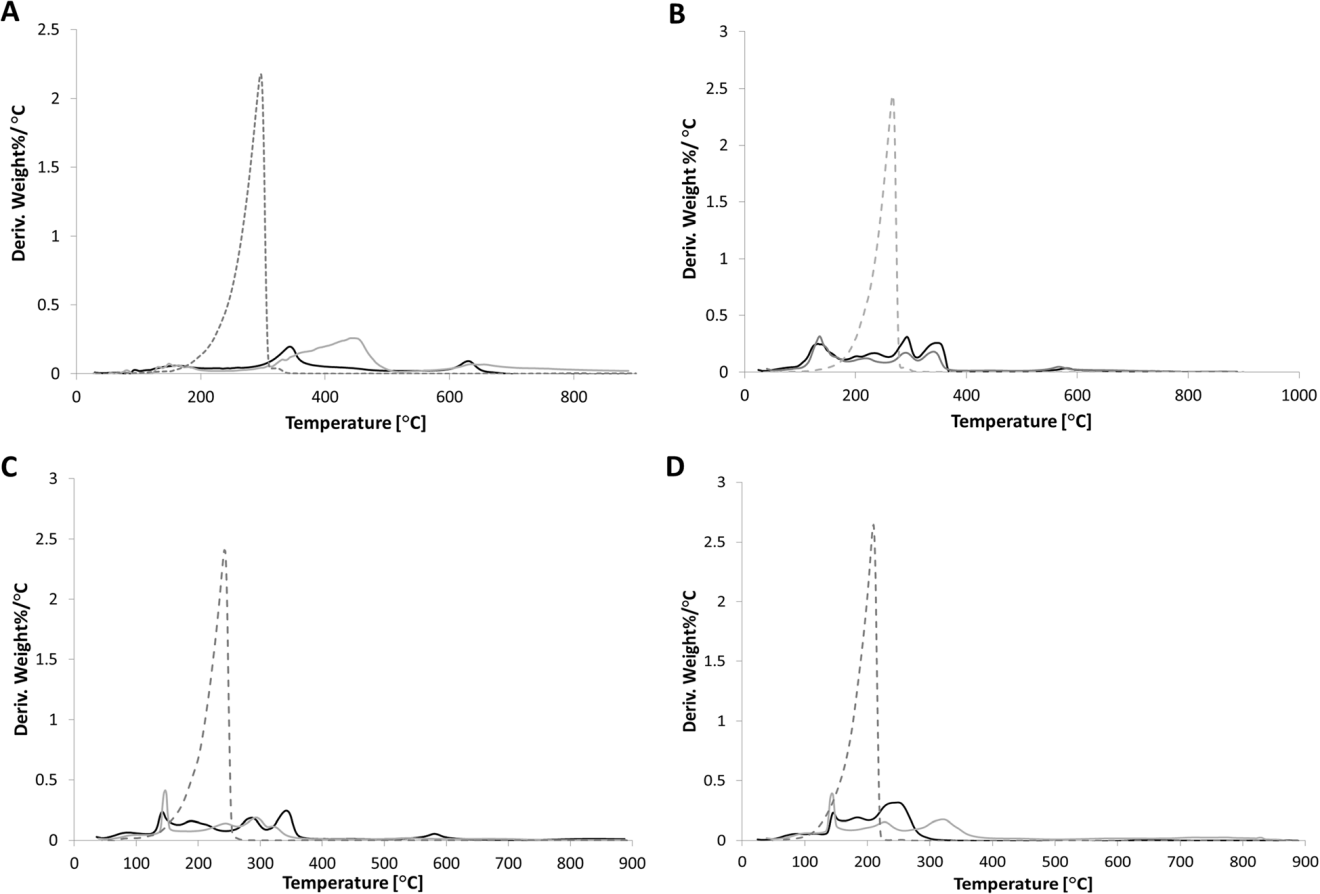
**DSC curves of SIPPs and fatty amines.** DSC curves for the SIPPs synthesized using ODA **(A)**, HDA **(B)**, TDA **(C)**, and DDA **(D)**. Dotted line = ligand only, black line = 30-min reflux, and gray line = 60-min reflux.

**Figure 5:**
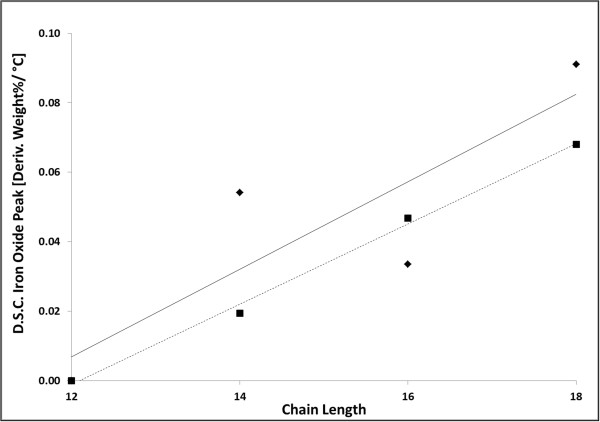
**Plot of DSC peak at approximately 600°C versus chain length.** Plot of the derivative weight percent per degrees Celsius for the iron oxide peak (approximately 580°C to 650°C) versus chain length. Diamond = solid line = 30-min reflux (*R*^2^ = 0.731). Square = dashed line = 60-min reflux (*R*^2^ = 0.996).

In addition to determining the size of the SIPPs and whether the ligands were bound to the surface, we also wanted to determine the composition of the SIPPs. We used TGA and DSC to determine the weight percent of ligand versus naked iron-platinum alloy. We also used the DSC capabilities in an attempt to characterize the amount, if any, of iron oxide contamination in the samples. Figure 3 shows the thermograms for each of the particles and fatty amines. The weight percent values of the ligands and naked iron-platinum are listed in Table 1 for each of the nanoparticles synthesized. In general, slightly more naked iron-platinum was found in the particles synthesized with the shorter-chained fatty amines, TDA, and DDA. Using the weight percent of ligands found using TGA, we calculated the number of ligands bound per milligram of SIPPs (Table 1). No significant differences were seen for the different preparations, and we calculated between 6.0 × 10^17^ and 1.3 × 10^18^ ligands per milligram of SIPPs. It has been reported that iron oxide contamination can be seen as a weight percent loss in the TGA thermograms around 590°C [14]. Although it is difficult to deduce anything in this temperature range from our thermograms, peaks are clearly apparent in the DSC plots of the derivative weight percent loss per degrees Celsius versus the temperature (Figure 4). Figure 4 shows clear peaks in the temperature range of 580°C to 650°C and may indicate iron oxide contamination in the samples. We plotted the intensity of these DSC peaks versus increasing chain length (Figure 5) and at 60-min reflux times, we found a very linear correlation between increasing chain length and increasing iron oxide contamination (*R*^2^ = 0.996). This linear correlation is not present with 30-min reflux times. This suggests that shorter reflux times reduce the amount of iron oxide contamination in the samples. Taken together with the TEM images and size analysis, this again indicates to us that the shorter chain fatty amine (TDA) is more efficient at making less polydispersed and pure (lower iron oxide contamination) SIPPs.

**Figure 6:**
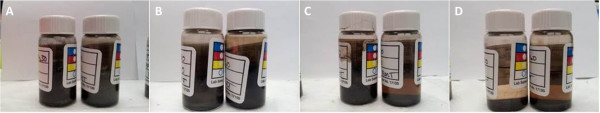
**Stability of SIPPs.** Suspensions of SIPPs synthesized using ODA **(A)**, HDA **(B)**, TDA **(C)**, and DDA **(D)** and allowed to reflux for either 30 or 60 min (left and right vials, respectively). Images were taken 1 week post-synthesis.

We next used ICP-OES to quantify the amount of iron and platinum in each of the samples. Moreover, we used this data to calculate the iron/platinum stoichiometry as well as the atomic percent of iron and platinum. The measured amounts of iron and platinum are listed in Table 1. It is evident that, in general, we saw increasing iron and platinum concentrations with increasing chain length. Also, except for the SIPPs synthesized with HDA, the atomic percent iron was fairly stable at approximately 50% regardless of the fatty amine used. Using the data generated thus far, we also calculated the particle volume, surface area, number of nanoparticles per milliliter of suspension, suspension concentration, and mass per particle to comprehensively characterize the structural properties of the samples. All of the structural characterizations are listed in Table 1. Stability is also an important factor in nanoparticle synthesis. Ideally, for mass production, nanoparticles should be stable for long periods of time in solution at room temperature. Figure 6 shows an image of the various SIPP preparations after sitting on the lab bench at room temperature for 1 week. The SIPPs made with the carbon-12 chain DDA fell out of the solution and were not stable. Similarly, the particles made with the carbon-14 chain TDA that were allowed to reflux for 60 min also fell out of solution in under 1 week at room temperature. Interestingly, the TDA-SIPPs that were only allowed to reflux for 30 min did not fall out of solution and were stable in solution at room temperature, as were all of the other particles prepared with ODA and HDA. All of the particles except the DDA-SIPPs and the 60-min refluxed TDA-SIPPs remained in solution for at least 3 months at room temperature, at which point we had used all of the samples.

**Figure 7:**
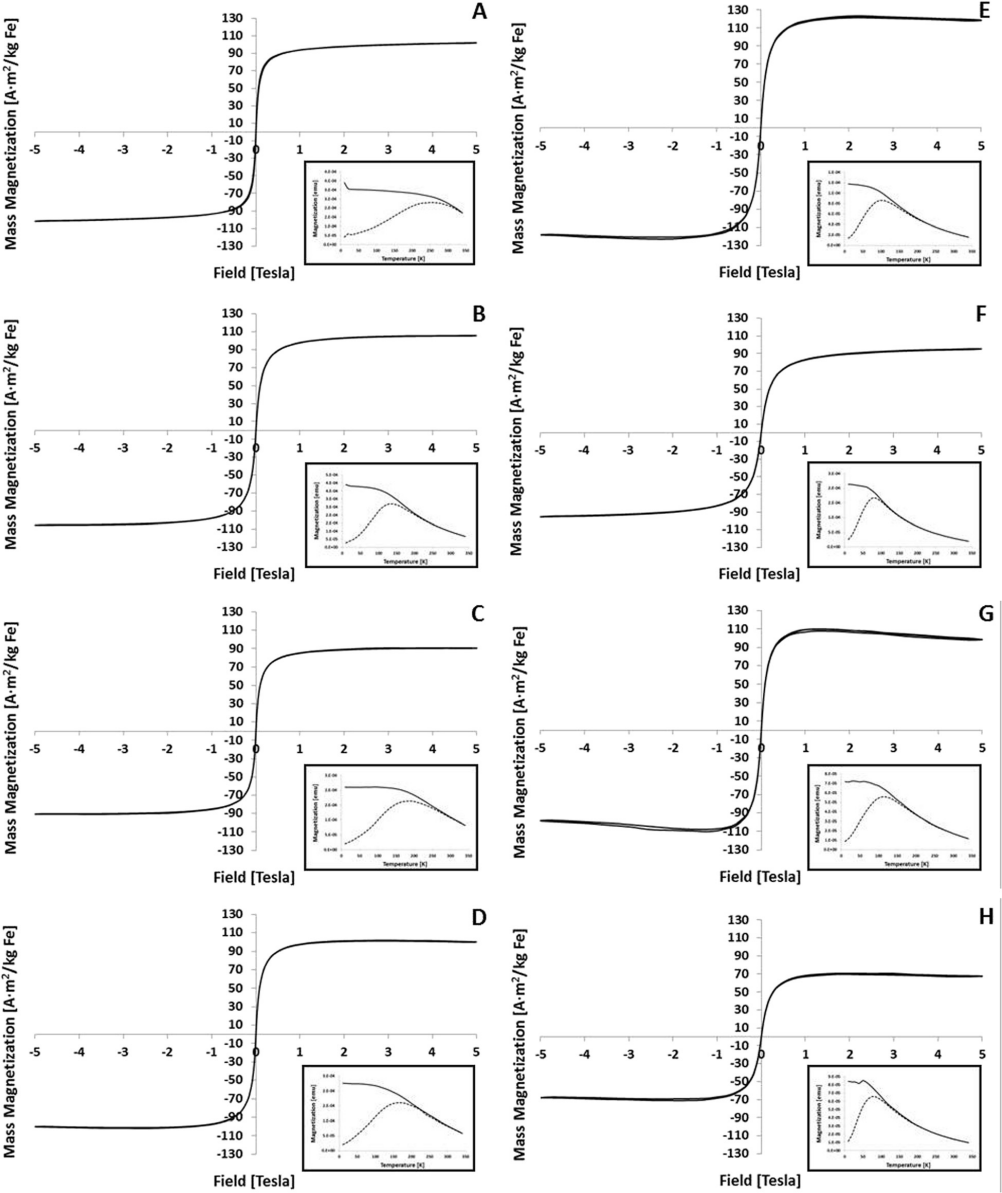
**Magnetic characteristics of SIPPs.** Aliquots (100 μL) of ODA-SIPPs **(A, B)**, HDA-SIPPs **(C, D)**, TDA-SIPPs **(E, F), **and DDA-SIPPs **(G, H)** were dried on Qtips® and measured using SQUID magnetometry. Hysteresis curves (M vs. H) are shown for SIPPs synthesized using either a 30-min **(A, C, E, G)** or 60-min **(B, D, F, H)** reflux time. The negative slope seen at high field is due to a diamagnetic contribution for the organic molecules (solvent and ligands). Insets show the ZFC (dashed line) and FC (solid line) curves for each of the SIPPs.

Upon fully characterizing the structural properties of the SIPPs, we aimed to measure the magnetic characteristics of the synthesized particles next. We used SQUID magnetometry to measure the saturation magnetization and blocking temperatures of each preparation of SIPPs. Figure 7 shows the hysteresis curves for each SIPP sample, as well as the ZFC/field-cooled (FC) curves. All of the samples had blocking temperature below room temperature, indicating that all of the particles are superparamagnetic. All of the samples had very high effective anisotropies and also had high mass magnetization between 71 A m^2^/kg iron and 123 A m^2^/kg iron. The highest saturation magnetization was measured for the carbon-14 TDA-SIPPs that were allowed to reflux for 30 min (123.39 A m^2^/kg iron). The magnetic characteristics are listed and compared in Table 2.

**Table 2:** Magnetic characterization of SIPPs

**Chain length**	**Reflux time (min)**	**Blocking temperature (K)**	**Saturation magnetization (A m**^**2**^**/kg iron)**	**Effective anisotropy (J/m**^**3**^**)**
18	30	255	101.93	4.5 × 10^4^
18	60	140	105.79	2.5 × 10^5^
16	30	190	90.79	3.9 × 10^5^
16	60	170	101.96	8.2 × 10^5^
14	30	100	123.39	1.7 × 10^5^
14	60	80	95.53	2.3 × 10^5^
12	30	110	110.24	1.5 × 10^5^
12	60	80	71.11	1.3 × 10^5^

After examining the various properties of the synthesized nanoparticles (high monodispersity, purity, stability, and high magnetic qualities), we believe that the carbon-14 TDA-SIPPs that were allowed to reflux for 30 min are superior nanoparticles compared with SIPPs synthesized using the other fatty amines. To confirm the validity of our findings, we repeated the synthesis of SIPPs using the fatty amine, TDA, in a 30-min reflux reaction. We fully characterized both structural and magnetic properties of the second batch of TDA-SIPPs and compared the results to those of the initial batch. Table 3 shows the comparison of the two different preparations of TDA-SIPPs. Reproducibility is seen in the size and shape of the TDA-SIPPs. Likewise, fairly good reproducibility is also seen for the other structural characteristics such as volume, surface area, concentration, and iron/platinum stoichiometry. Table 4 compares the magnetic characterizations of the two separate TDA-SIPP preparations. Again, the reproducibility is fairly good, and the particles had similar blocking temperatures and mass magnetizations. The average mass magnetization of the TDA-SIPPs was 108.98 A m^2^/kg iron ± 20.38 A m^2^/kg iron. This value of mass magnetization was still higher than that measured for the other SIPPs made with all of the other fatty amines examined in this study (DDA, HDA, and ODA).

**Table 3:** Comparison of SIPPs made with tetradecylamine and a 30-min reflux

**Value**	**Description**	**Units**	**TDA-SIPP no. 1**	**TDA-SIPP no. 2**
*d*	Diameter	nm	7.34 ± 1.22	7.86 ± 0.76
CV	Coefficient of variation	%	16.6	9.6
*V*_p_	Particle volume	cm^3^	2.07 × 10^−19^	2.55 × 10^−19^
*S*	Surface area	cm^2^	1.69 × 10^−12^	1.94 × 10^−12^
*C*_p_	Suspension concentration	mg/mL	4.29 ± 0.47	5.97 ± 0.14
*C*_Fe_	Iron concentration	mg/mL	0.214 ± 0.00007	0.729 ± 0.004
*C*_Pt_	Platinum concentration	mg/mL	0.583 ± 0.0003	2.503 ± 0.005
*N*^a^_Fe_	Iron atoms in 1.0 mL	-	2.31 × 10^18^	7.87 × 10^18^
*N*_SIPP_	Nanoparticles per mL	SIPP/mL	5.90 × 10^14^	1.83 × 10^15^
*A*_Fe_	Atomic percent Fe	at.%	56.2	50.4
*A*_Pt_	Atomic percent Pt	at.%	43.8	49.6
Fe/Pt	Fe/Pt stoichiometry	-	1.28	1.02
*M*^P^_FePt_	Mass per particle	g	2.9 × 10^−18^	3.56 × 10^−18^
*N*^a^_FePt_	Total Fe + Pt atoms per particle	-	6,964	8,551
*N*^P^_Fe_	Iron atoms per particle	-	3913.8	4309.9
*N*^P^_Pt_	Platinum atoms per particle	-	3050.3	4241.5

**Table 4:** **Average magnetic properties of TDA-SIPPs (*n*** **= 2)**

**Value**	**Description**	**Units**	**TDA-SIPP no. 1**	**TDA-SIPP no. 2**	**Mean**
*T*_b_	Blocking temperature	K	100	150	125 ± 35.3
*M*_sat_	Saturation magnetization	A m^2^/kg iron	123.39	94.57	108.98 ± 20.38
*K*	Effective anisotropy	J/m^3^	1.7 × 10^5^	2.0 × 10^5^	1.8 × 10^5^ ± 2.6 × 10^4^

## Conclusions

Iron-platinum particles were successfully synthesized using four different fatty amines, from 12 to 18 carbons in length. Although some iron oxide contamination was seen, this decreased with increasing reflux time and decreasing chain length. Additionally, increasing the amount of time that the particles were allowed to reflux also increased the diameter of the particles, but decreased the iron concentration. This was due to the reduction in the iron to platinum stoichiometry that was measured in all of the 60-min reflux reactions, compared to the reactions allowed to reflux for only 30 min. Again, this indicates that shorter reaction times are preferable. SIPPs synthesized using DDA were the least stable in addition to being corrosive to the reflux apparatus. We found that using TDA and a 30-min reflux reaction created the optimal particles with the highest degree of monodispersity, iron content, and stability. There have been several reports of using SIPPs for *in vivo* applications [2,15-17]. Uniformity of size and shape of nanoparticles are important for issues related to biocompatibility, as a widely varying size range may lead to non-uniform behavior of the nanoparticles both *in vitro* and *in vivo*. Moreover, for applications involving magnetic resonance imaging (MRI) for cancer detection, a high magnetic moment is preferable, as this correlates with a higher contrast enhancement in the magnetic resonance images. Our synthesized TDA-SIPPs show higher degree of monodispersity, as well as higher saturation magnetizations compared to other SIPPs previously reported in the literature [8-10]. Therefore, SIPPs synthesized using TDA could be useful not only due to their ‘greener’ method of synthesis and ease of scaling up the synthesis but also as potentially better MRI contrast agents for cancer detection. Our novel finding in the current study is different compared to those in the current literature where octadecylamine is the preferred ligand most commonly used for the routine synthesis of SIPPs [8-10,15,16].

## Abbreviations

ATR: attenuated total reflectance; DDA: dodecylamine; DDA-SIPPs: SIPPs synthesized with the ligand DDA; DSC: differential scanning calorimetry; EtOH: ethanol; FC: field cooled; FTIR: Fourier transform infrared spectroscopy; HDA: hexadecylamine; HDA-SIPPs: SIPPs synthesized with the ligand HDA; ICP-OES: inductively coupled plasma-optical emission spectroscopy; ODA: octadecylamine; ODA-SIPPs: SIPPs synthesized with the ligand ODA; ODE: octadecene; SIPPs: superparamagnetic iron platinum particles; SQUID: superconducting quantum interference device; TCA: tetracosane; TDA: tetradecylamine; TDA-SIPPs: SIPPs synthesized with the ligand TDA; TEM: transmission electron microscopy; TGA: thermogravimetric analysis; ZFC: zero-field cooled.

## Competing interests

The authors declare that they have no competing interests.

## Authors' contributions

RMT designed the study, acquired, analyzed, and interpreted the data, and drafted the manuscript. TCM acquired and analyzed data and helped draft the manuscript. RRG conceived and designed the study, interpreted the data, and drafted the manuscript. All authors read and approved the final manuscript.
